# Administration of Myelin Basic Protein Peptides Encapsulated in Mannosylated Liposomes Normalizes Level of Serum TNF-*α* and IL-2 and Chemoattractants CCL2 and CCL4 in Multiple Sclerosis Patients

**DOI:** 10.1155/2016/2847232

**Published:** 2016-04-28

**Authors:** Yakov Lomakin, Alexey Belogurov, Irina Glagoleva, Alexey Stepanov, Konstantin Zakharov, John Okunola, Ivan Smirnov, Dmitry Genkin, Alexander Gabibov

**Affiliations:** ^1^Institute of Bioorganic Chemistry RAS, Moscow 117997, Russia; ^2^Institute of Fundamental Medicine and Biology, Kazan Federal University, Kazan 420012, Russia; ^3^Institute of Gene Biology RAS, Moscow 119334, Russia; ^4^OJSC Pharmsynthez, Saint Petersburg 197110, Russia; ^5^Chemistry Department, Moscow State University, Moscow 119991, Russia

## Abstract

We have previously shown that immunodominant MBP peptides encapsulated in mannosylated liposomes (Xemys) effectively suppressed experimental allergic encephalomyelitis (EAE). Within the frames of the successfully completed phase I clinical trial, we investigated changes in the serum cytokine profile after Xemys administration in MS patients. We observed a statistically significant decrease of MCP-1/CCL2, MIP-1*β*/CCL4, IL-7, and IL-2 at the time of study completion. In contrast, the serum levels of TNF-*α* were remarkably elevated. Our data suggest that the administration of Xemys leads to a normalization of cytokine status in MS patients to values commonly reported for healthy subjects. These data are an important contribution for the upcoming Xemys clinical trials.

## 1. Introduction

Multiple sclerosis (MS) is an autoimmune neurologic disease with unclarified etiology. It is well established that the proliferation of autoreactive T and B cells leads to CNS inflammation and subsequent myelin destruction. CD4+ T cells are believed to be the essential mediators of the inflammatory process in MS. However, the role of B cells, macrophages, and T cells other than CD4+ should not be neglected [1]. Almost all chronic inflammatory diseases and immunopathologies are linked to dysregulated cytokine responses; thus it is evident that cytokine status plays an important role in MS progression [2, 3]. Cytokines and chemokines not only suppress or activate different types of immune cells, but also can facilitate the migration of pathogenic cells through the blood-brain barrier (BBB) [4].

There are a large number of studies describing cytokine status by direct analysis in the blood and cerebrospinal fluid (CSF) [5, 6] and determining mRNA expression in peripheral blood mononuclear cells (PBMCs) of MS patients [7, 8]. Generally, it is believed that the immune response in MS is shifted towards Th1 cytokine production (IL-6, IL-12, IL-2, IFN-*γ*, and TNF-*α*). The most notable study of cytokine involvement in MS progression involved the administration of interferons in humans. While IFN-*β* is fully approved [9, 10] and appears to be one of the most common treatments for MS worldwide the injection of IFN-*γ* threatens the health and life of MS patients [11].

Along with the physiological assessment, the level of cytokines may be an important diagnostic criterion [12]. During clinical trials, it is a common practice to analyze cytokine levels in different physiological fluids, particularly in terms of the Th1/Th2 ratio; this analysis may shed light on disease pathogenesis, mechanism of drug action and methods of their improvement, and modifications in administration strategy. It was shown that after long-term IFN-*β* therapy, the serum concentration of IFN-*γ* decreased [13]. Others have shown that treatment with IFN-*β* results in increased levels of TNF-*α* and decreased levels of IL-5. Additionally, the combination of IFN-*β* administration with atorvastatin therapy increases the levels of proinflammatory cytokine IL-12p70 [14]. A significantly elevated Th2/Th1 ratio was shown to be a hallmark of glatiramer acetate administration [15]. Recently, a similar effect was shown in MS patients treated with monoclonal anti-*α*4-integrin antibody (natalizumab) [16].

Previously, we reported the efficiency of immunodominant MBP peptides encapsulated in mannosylated liposomes (Xemys) in suppressing experimental allergic encephalomyelitis (EAE) [17, 18]. This therapeutic composition was created in parallel with a variety of techniques to induce tolerance towards MBP [19–24]. Our study demonstrated that the uptake of distinct MBP peptides, which are immunodominant in terms of autoantibody response in MS patients [25], by dendritic cells was enhanced by mannosylation of carrier liposomes. In frames of successfully completed phase I clinical studies, we analyzed the serum cytokine profile in MS patients treated with Xemys. The aim of this study was to characterize changes in cytokine levels and the Th1/Th2 ratio after Xemys administration.

## 2. Materials and Methods

### 2.1. Patients

Phase I included patients with relapse-remitting MS (RRMS) or secondary progressive MS (SPMS) according to the McDonald diagnostic criteria in 2005 [26]. A total of 18 MS patients were subcutaneously administered with a total of 2.675 mg of encapsulated MBP peptides. Patients were enrolled to receive weekly subcutaneous injection (s.c.) of Xemys at increasing doses from 50 *μ*g to 900 *μ*g over six weeks. All patients had a follow-up at 6–18 weeks. The study was authorized by the Russian Public Health Ministry #930 [FASEMS-01/01], which was issued on April 28, 2012. All patients provided written informed consent at the time of enrollment.

### 2.2. Profiling of Serum Cytokines

A cytokine profile analysis was performed with serum samples (collected at baseline and during all follow-up visits) using a multiplexed fluorescent magnetic bead-based immunoassay (Bio-Rad Laboratories, USA) according to the manufacturer's instructions. The following 17 different cytokines and chemokines were assessed: IL-1*β*, IL-2, IL-4, IL-5, IL-6, IL-7, IL-8, IL-10, IL-12 (p70), IL-13, IL-17A, granulocyte colony-stimulating factor (G-CSF), granulocyte macrophage colony-stimulating factor (GM-CSF), IFN-*γ*, monocyte chemoattractant protein-1 (MCP-1/CCL2), macrophage inflammatory protein (MIP-1*β*/CCL4), and tumor necrosis factor-alpha (TNF-*α*). All samples were measured in triplicate. For the baseline, the cytokine and chemokine levels in healthy subjects (median and IQR) from previously published data were used for CCL2, CCL4, IL-6, IL-7, IL-10, IL-12, G-CSF [27], IL-8, IL-17, TNF-*α* [28], IFN-*γ* [29], IL-4 [30], IL-13 [31], IL-5 [32], and IL-2 (http://www.quanterix.com/files/assays/IL-2%20Data%20Sheet%20ID%204904%20011615.pdf).

### 2.3. Statistical Analysis

Data were analyzed by using the Sigma-Plot 12.5 and Statistica 10 software. The difference in the cytokine levels before and after treatment was compared by using Student's *t*-test, nonparametric Mann-Whitney *U* test, and Wilcoxon signed-rank test. If the measured values were under the detection limit, they were considered to be equal to the value of the detection limit. A two-sided *p* value < 0.05 was considered statistically significant. The difference was considered reliable when confirmed by at least one test. 

## 3. Results and Discussion

Phase I Xemys clinical studies involved 16 (80%) patients with RRMS and 4 (20%) patients with SPMS with relapses. Baseline characteristics of MS patients are listed in Table 1. Three (15%) patients had a mild disability according to the expanded disability status scale (EDSS) (3.0) and 17 (85%) patients showed moderate disability (3.5–5.5) at baseline. Nineteen MS patients were subcutaneously administered a total of 2.675 mg encapsulated MBP peptides, which was divided into 6 injections with increasing dose every week (weeks 1–6). One patient received only the initial 50 *μ*g dose of Xemys and was discontinued from the study during the first week of the treatment period. A complete set of serum samples was obtained for 18 patients. To analyze the immunological consequences of Xemys administration, the levels of 17 serum cytokines and chemokines were analyzed at baseline (week −2) and during the follow-ups (weeks 7, 10, and 18) (Table 2).

**Figure 1:**
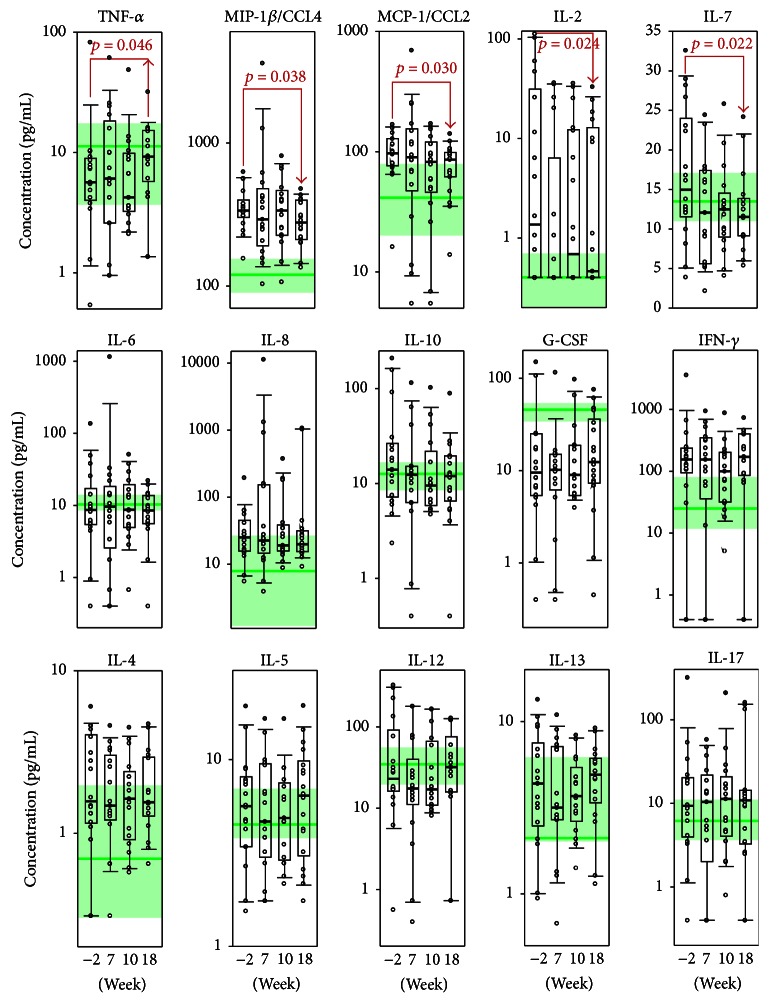
Serum levels of cytokines (pg/mL) in the treatment group at the time of the screening (−2 weeks), 7, 10, and 18 weeks. The interquartile range is shown by boxes. The median in each group is shown by the bold line. Bars represent 95% confidence interval. Statistically significant differences with their respective *p* values are indicated. The green line represents cytokine levels in healthy individuals. The green-colored zone corresponds to the interquartile range in healthy individuals according to the previously reported data (see Section 2 for details). In the upper line, cytokines that had a statistically significant improvement are depicted.

As anticipated, in comparison to healthy individuals, MS patients at baseline revealed increased levels of proinflammatory cytokines and chemokines IFN-*γ*, IL-2, IL-8, IL-17, CCL2, and CCL4 whereas level of anti-inflammatory cytokines IL-4, IL-10, and IL-13 was not significantly dysregulated. These data partially correlate with previously reported data [3] demonstrating increased level of IL-12, IFN-*γ*, IL-17, and CCL4 in cerebrospinal fluid (CSF) of MS patients. Interestingly, level of CCL2 in opposite was shown to decrease in CSF during MS. In the present study, a statistically significant decrease was observed in the serum levels of MCP-1/CCL2, MIP-1*β*/CCL4, IL-7, and IL-2 at the time of study completion (week 18) (Figure 1). Importantly, the median level of all effector molecules, except IL-7, was outside of the interquartile range that corresponded to the healthy subjects, suggesting that they were significantly upregulated in MS patients. Our data are in accordance with studies reporting that both CCL2 [33] and CCL4 [34, 35] are elevated during the course of MS. These chemokines recruit monocytes, memory T cells, and dendritic cells to the sites of inflammation, across the BBB and within the CNS parenchyma [36]. Cheng et al. reported that treatment with IFN-*β* results in decreased CCL2 and CCL4 levels in the CNS of C57BL/6 mice inflicted with EAE [37]. The mechanism is complicated for CCL2 in MS because it is elevated only in the blood and is decreased in the CSF; this likely involves the action of CCR2-positive migrating cells as they cross the BBB [38].

Previously, we reported that treatment of DA rats with encapsulated MBP peptides resulted in a downregulation of the proinflammatory cytokine IL-2 in the CNS [18]. In line with this, the levels of serum IL-2 were decreased in MS patients after Xemys treatment. It should be mentioned that, for 40% of patients (7/18), IL-2 concentrations were under the detection limit (0.4 pg/mL) at all follow-up time points, while for the rest of the patients, except for 1, elevated IL-2 levels returned to the levels of healthy subjects after Xemys administration.

IL-7 is a cytokine that is important for B and T cell development [39]. This cytokine forms a heterodimer with the hepatocyte growth factor (HGF) and the heterodimer functions as a pre-pro-B cell growth-stimulating factor [40]. In contrast, Lee et al. showed that increased IL-7 in the serum is a hallmark of the Th1-driven form of MS, and the blockade of IL-7 and the IL-7R*α* pathway may have a therapeutic potential in MS [41]. Thus, the observed downregulation of IL-7 after Xemys treatment may be therapeutically beneficial.

Our data suggest that the levels of serum TNF-*α* are elevated after Xemys administration (Figure 1). This statistically significant observation may have important physiological relevance. Interestingly, TNF-*α* is considered to be a potent mediator of inflammation in MS [42]. It should be noted that, in the CSF, but not in the serum of MS patients, TNF-*α* levels are significantly higher and correlates with the severity and progression of the disease [43]. However, it was suggested that TNF-*α* is a potent anti-inflammatory cytokine in autoimmune demyelination [44, 45] and TNF-*α*-related genes are downregulated during MS progression [8]. Clinical studies with TNF-*α*-inhibiting agents revealed an increased frequency of MS relapse [46–48]. Additional evidence to show that TNF-*α* influences MS could be that a shorter TNF-*α* receptor 1 with two “A” instead of two “G” increases the probability of MS development to 12% by mimicking the effect of TNF-blocking drugs [49, 50].

Serum concentrations of IL-8, IL-6, and IL-10 decreased to the levels of healthy subjects without any statistically significant differences (Figure 1). This observation is promising because previous studies reported that elevated levels of these cytokines were associated with MS progression. PBMC gene expression analysis revealed that IL-8 was significantly overexpressed in patients with MS and other autoimmune diseases [51]. Moreover, the levels of IL-8 are elevated in the CSF of MS patients [52]. Correlative analysis between the cytokine profile and the progression of the disease [29] revealed that the levels and ranges of IL-6 are elevated in MS patients and this increases with disease severity [29]. Our data showed that the median value of IL-6 had not changed because only a few cases showed elevated levels for this cytokine and all levels were restored to normal levels of healthy subjects upon study completion. Recently published data report anti-inflammatory activities of IL-6 protecting mice from organ specific autoimmune disease by IL-6 classical signalling-dependent IL-1ra induction [53]. Thus IL-6 may be potentially considered as beneficial in combination with Xemys. IL-10 was slightly elevated in MS patients, but there was no correlation with disease severity [29]. The other cytokine levels were unchanged, while the level of GM-CSF was under the detection limit.

**Figure 2:**
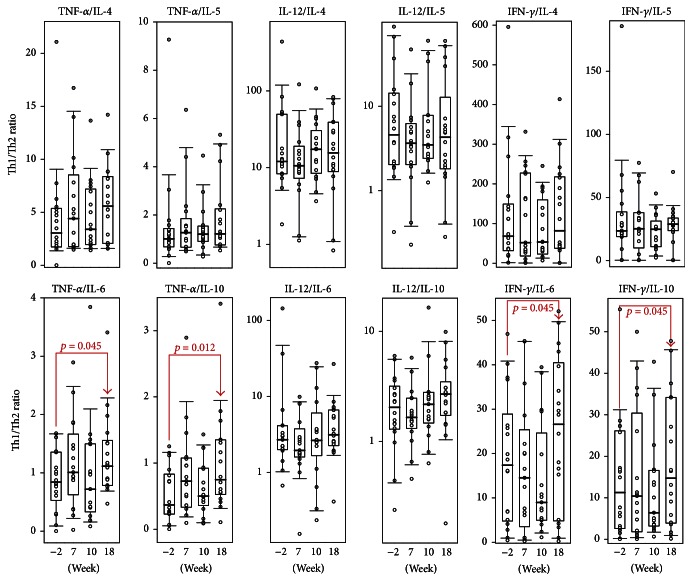
The Th1/Th2 serum ratio in the treatment group at the time of the screening (−2 weeks), 7, 10, and 18 weeks. The interquartile range is shown by boxes. The median in each group is shown by the bold line. Bars represent 95% confidence interval. Statistically significant differences with their respective *p* values are indicated.

The balance between Th1 and Th2 cytokines is very important as they can functionally cross-inhibit each other. MS is considered to be a Th1-mediated disorder; however number of studies argue with this [54–56]. Th1 cells and their related activation pathways are linked to the production of IFN-*γ* and, to a lesser extent, with IL-12, IL-2, and TNF-*α*. Th2 cells are heavily reliant on IL-4, IL-5, and IL-10. Therefore, we defined the Th1/Th2 ratio between IFN-*γ*, TNF-*α*, and IL-12 towards IL-4, IL-5, IL-6, and IL-10 (Figure 2). Interestingly, the administration of Xemys shifted the Th1/Th2 ratio towards mostly Th1. The observed shift may be due to the downregulation of the Th2 cytokines IL-6 and IL-10 and an elevated production of TNF-*α* rather than by a systemic elevation of Th1 cytokines.

## 4. Conclusions

In terms of serum cytokines, a reduction of inflammation and restriction of monocyte cell trafficking are immunological consequences of the administration of encapsulated MBP peptides in MS patients. In the present study, we found that cytokine levels returned to normal levels of healthy subjects after Xemys treatment in MS patients, especially for IL-2, IL-7, CCL2, CCL4, and TNF-*α*. However, the levels of IFN-*γ* were unchanged and significantly higher than healthy subjects. The administration of IFN-*β* decreased IFN-*γ* secretion and inhibited IFN-*γ*-related responses in MS patients [57–59]. Therefore, concomitant therapy utilizing Xemys in combination with an anti-IFN-*γ* treatment [60] may be more beneficial. This investigation is preliminary and definitive conclusions could not be made because the cohort is limited and heterogeneous, the follow-up period was short, and the placebo group was absent. Nonetheless, the observed changes in serum cytokines and chemokines may be important to consider for the upcoming Xemys phase II clinical trial.

## Figures and Tables

**Table 1:** Patients' baseline characteristics.

Characteristic	MS patients
*N*	20
Age (yrs.)^a^	37.6 ± 9.9 (24–53)
Gender^b^	9–11 (45%)
Weight (kg)^a^	69.7 ± 15.8 (44–105.5)
First MS symptoms (yrs.)^a^	10.4 ± 6.8 (0.9–25.8)
MS diagnosed (yrs.)^a^	5.0 ± 3.7 (0.6–13.3)
EDSS^c^	4.0 (3.0–5.5)
Relapse remitting^d^	16 (80.0%)
Secondary progressive^d^	4 (20.0%)
Total relapses (1 yr.)^c^	2.0 (1–3)
Total relapses (2 yrs.)^c^	3.0 (1–6)

^a^Average ± standard deviation (range).

^b^Female-male (female%).

^c^Median (range).

^d^Number (%).

**Table 2:** Level of serum cytokines and chemokines (pg/mL) in MS patients at baseline and follow-up period.

Cytokine	−2 weeks		7 weeks		10 weeks		18 weeks	
	Median	IQR	Median	IQR	Median	IQR	Median	IQR
TNF-*α*	5.7	4.2–8.7	6.1	2.6–15.9	4.2	3.4–9.3	9.2	6.6–14.5
IFN-*γ*	155	107–226	155	58–332	100	33–203	171	91–360
CCL4	337	311–378	293	208–429	337	229–441	278	216–394
CCL2	97	78–123	90	57–142	82	49.3–111	86	64–98
G-CSF	9.5	5.7–23.2	10.2	7.2–14.6	9.0	5.6–18.6	12	8–32
IL-1*β*	2.5	2.4–4.0	3.6	2.9–6.4	2.9	2.3–3.2	3.2	3.1–3.7
IL-2	1.4	0.4–22.4	0.4	0.4–1.6	0.7	0.4–10.7	0.5	0.4–10
IL-4	1.6	1.2–3.7	1.5	1.3–2.8	1.6	1.0–2.2	1.6	1.3–2.6
IL-5	5.6	3.6–7.8	4.7	3.2–9.3	4.9	3.0–7.2	6.4	3.4–9.2
IL-6	8.6	5.6–14.3	9.6	3.4–14.8	8.7	5.1–16.6	8.5	5.7–13.3
IL-7	15	12–23	12.1	5.8–17.2	12.5	9.4–13.6	11.5	9.2–13.6
IL-8	25	16–39	23	17–146	19	16–35	20	16–30
IL-10	14	7–25	12.4	6.3–15.2	9.5	6.3–16.8	12	7–19
IL-12	23	17–67	18	13–28	17	11–60	32	16–57
IL-13	4.4	2.7–6.8	3.2	2.7–7.0	3.7	2.9–4.8	4.9	3.6–6.0
IL-17	9.3	4.6–19.7	10.4	3.6–18.8	11.3	4.6–17.1	11	3.9–14.1
GM-CSF		Under detection limit						

## References

[1] Nylander A., Hafler D. A. (2012). Multiple sclerosis. *Journal of Clinical Investigation*.

[2] Aarli J. A. (2003). Role of cytokines in neurological disorders. *Current Medicinal Chemistry*.

[3] Kothur K., Wienholt L., Brilot F., Dale R. C. (2016). CSF cytokines/chemokines as biomarkers in neuroinflammatory CNS disorders: a systematic review. *Cytokine*.

[4] Cheng W., Chen G. (2014). Chemokines and chemokine receptors in multiple sclerosis. *Mediators of Inflammation*.

[5] Maimone D., Gregory S., Arnason B. G. W., Reder A. T. (1991). Cytokine levels in the cerebrospinal fluid and serum of patients with multiple sclerosis. *Journal of Neuroimmunology*.

[6] Mouzaki A., Rodi M., Dimisianos N. (2015). Immune parameters that distinguish multiple sclerosis patients from patients with other neurological disorders at presentation. *PLoS ONE*.

[7] Nejati A., Shoja Z., Shahmahmoodi S. (2016). EBV and vitamin D status in relapsing-remitting multiple sclerosis patients with a unique cytokine signature. *Medical Microbiology and Immunology*.

[8] Achiron A., Gurevich M., Friedman N., Kaminski N., Mandel M. (2004). Blood transcriptional signatures of multiple sclerosis: unique gene expression of disease activity. *Annals of Neurology*.

[9] Paty D. W., Li D. K. (1993). Interferon beta-1b is effective in relapsing-remitting multiple sclerosis. II. MRI analysis results of a multicenter, randomized, double-blind, placebo-controlled trial. UBC MS/MRI Study Group and the IFNB Multiple Sclerosis Study Group. *Neurology*.

[10] PRISMS (Prevention of Relapses and Disability by Interferon β-1a Subcutaneously in Multiple Sclerosis) Study Group (1998). Randomised double-blind placebo-controlled study of interferon *β*-1a in relapsing/remitting multiple sclerosis. *The Lancet*.

[11] Panitch H. S., Hirsch R. L., Schindler J., Johnson K. P. (1987). Treatment of multiple sclerosis with gamma interferon: exacerbations associated with activation of the immune system. *Neurology*.

[12] Dimisianos N., Rodi M., Kalavrizioti D., Georgiou V., Papathanasopoulos P., Mouzaki A. (2014). Cytokines as biomarkers of treatment response to IFN*β* in relapsing-remitting multiple sclerosis. *Multiple Sclerosis International*.

[13] Petek-Balci B., Çoban A., Shugaiv E. (2015). Predictive value of early serum cytokine changes on long-term interferon beta-1a efficacy in multiple sclerosis. *The International Journal of Neuroscience*.

[14] Sellner J., Greeve I., Findling O. (2008). Effect of interferon-*β* and atorvastatin on Th1/Th2 cytokines in multiple sclerosis. *Neurochemistry International*.

[15] Neuhaus O., Farina C., Yassouridis A. (2000). Multiple sclerosis: Comparison of copolymer-1-reactive T cell lines from treated and untreated subjects reveals cytokine shift from T helper 1 to T helper 2 cells. *Proceedings of the National Academy of Sciences of the United States of America*.

[16] Oreja-Guevara C., Ramos-Cejudo J., Aroeira L. S., Chamorro B., Diez-Tejedor E. (2012). TH1/TH2 Cytokine profile in relapsing-remitting multiple sclerosis patients treated with Glatiramer acetate or Natalizumab. *BMC Neurology*.

[17] Belogurov A. A., Zargarova T. A., Turobov V. I. (2009). Suppression of ongoing experimental allergic encephalomyelitis in da rats by novel peptide drug, structural part of human myelin basic protein 4662. *Autoimmunity*.

[18] Belogurov A. A., Stepanov A. V., Smirnov I. V. (2013). Liposome-encapsulated peptides protect against experimental allergic encephalitis. *The FASEB Journal*.

[19] Garren H., Robinson W. H., Krasulová E. (2008). Phase 2 trial of a DNA vaccine encoding myelin basic protein for multiple sclerosis. *Annals of Neurology*.

[20] Katsara M., Deraos G., Tselios T. (2009). Design and synthesis of a cyclic double mutant peptide (cyclo(87-99)[A 91,A96]MBP87-99) induces altered responses in mice after conjugation to mannan: implications in the immunotherapy of multiple sclerosis. *Journal of Medicinal Chemistry*.

[21] Katsara M., Yuriev E., Ramsland P. A. (2008). A double mutation of MBP(83-99) peptide induces IL-4 responses and antagonizes IFN-gamma responses. *Journal of Neuroimmunology*.

[22] Peschl P., Reindl M., Schanda K., Sospedra M., Martin R., Lutterotti A. (2015). Antibody responses following induction of antigen-specific tolerance with antigen-coupled cells. *Multiple Sclerosis Journal*.

[23] Lutterotti A., Yousef S., Sputtek A. (2013). Antigen-specific tolerance by autologous myelin peptide-coupled cells: a phase 1 trial in multiple sclerosis. *Science Translational Medicine*.

[24] Liu X., Ciumas C., Huang Y.-M. (2005). Autoantigen-pulsed dendritic cells constitute a beneficial cytokine and growth factor network in ameliorating experimental allergic encephalomyelitis. *Multiple Sclerosis*.

[25] Belogurov A. A., Kurkova I. N., Friboulet A. (2008). Recognition and degradation of myelin basic protein peptides by serum autoantibodies: novel biomarker for multiple sclerosis. *The Journal of Immunology*.

[26] Polman C. H., Reingold S. C., Edan G. (2005). Diagnostic criteria for multiple sclerosis: 2005 revisions to the ‘McDonald Criteria’. *Annals of Neurology*.

[27] Kleiner G., Marcuzzi A., Zanin V., Monasta L., Zauli G. (2013). Cytokine levels in the serum of healthy subjects. *Mediators of Inflammation*.

[28] Arican O., Aral M., Sasmaz S., Ciragil P. (2005). Serum levels of TNF-*α*, IFN-*γ*, IL-6, IL-8, IL-12, IL-17, and IL-18 in patients with active psoriasis and correlation with disease severity. *Mediators of Inflammation*.

[29] Kallaur A. P., Oliveira S. R., Simao A. N. C. (2013). Cytokine profile in relapsing-remitting Multiple sclerosis patients and the association between progression and activity of the disease. *Molecular Medicine Reports*.

[30] Font-Ribera L., Kogevinas M., Zock J.-P. (2010). Short-term changes in respiratory biomarkers after swimming in a chlorinated pool. *Environmental Health Perspectives*.

[31] Spadaro A., Rinaldi T., Riccieri V., Valesini G., Taccari E. (2002). Interleukin 13 in synovial fluid and serum of patients with psoriatic arthritis. *Annals of the Rheumatic Diseases*.

[32] Joseph J., Benedict S., Safa W., Joseph M. (2004). Serum interleukin-5 levels are elevated in mild and moderate persistent asthma irrespective of regular inhaled glucocorticoid therapy. *BMC Pulmonary Medicine*.

[33] Van Der Voorn P., Tekstra J., Beelen R. H. J., Tensen C. P., Van Der Valk P., De Groot C. J. A. (1999). Expression of MCP-1 by reactive astrocytes in demyelinating multiple sclerosis lesions. *The American Journal of Pathology*.

[34] Tejera-Alhambra M., Casrouge A., de Andrés C. (2015). Plasma biomarkers discriminate clinical forms of multiple sclerosis. *PLoS ONE*.

[35] Matsushita T., Tateishi T., Isobe N. (2013). Characteristic cerebrospinal fluid cytokine/chemokine profiles in neuromyelitis optica, relapsing remitting or primary progressive multiple sclerosis. *PLoS ONE*.

[36] Tran P. B., Miller R. J. (2003). Chemokine receptors: signposts to brain development and disease. *Nature Reviews Neuroscience*.

[37] Cheng W., Zhao Q., Xi Y. (2015). IFN-*β* inhibits T cells accumulation in the central nervous system by reducing the expression and activity of chemokines in experimental autoimmune encephalomyelitis. *Molecular Immunology*.

[38] Mahad D., Callahan M. K., Williams K. A. (2006). Modulating CCR2 and CCL2 at the blood-brain barrier: relevance for multiple sclerosis pathogenesis. *Brain*.

[39] Morrissey P. J., Goodwin R. G., Nordan R. P. (1989). Recombinant interleukin 7, preB-cell growth factor, has costimulatory activity on purified mature T cells. *Journal of Experimental Medicine*.

[40] Lai L., Goldschneider I. (2001). Cutting edge: identification of a hybrid cytokine consisting of IL-7 and the *β*-chain of the hepatocyte growth factor/scatter factor. *The Journal of Immunology*.

[41] Lee L.-F., Axtell R., Tu G. H. (2011). IL-7 promotes T_H_1 development and serum IL-7 predicts clinical response to interferon-*β* in multiple sclerosis. *Science Translational Medicine*.

[42] Link H. (1998). The cytokine storm in multiple sclerosis. *Multiple Sclerosis*.

[43] Sharief M. K., Hentges R. (1991). Association between tumor necrosis factor-*α* and disease progression in patients with multiple sclerosis. *The New England Journal of Medicine*.

[44] Liu J., Marino M. W., Wong G. (1998). TNF is a potent anti-inflammatory cytokine in autoimmune-mediated demyelination. *Nature Medicine*.

[45] Mohan N., Edwards E. T., Cupps T. R. (2001). Demyelination occurring during anti-tumor necrosis factor *α* therapy for inflammatory arthritides. *Arthritis and Rheumatism*.

[46] (1999). TNF neutralization in MS: results of a randomized, placebo-controlled multicenter study: the Lenercept Multiple Sclerosis Study Group and The University of British Columbia MS/MRI Analysis Group. *Neurology*.

[47] Li S. Y., Birnbaum A. D., Goldstein D. A. (2010). Optic neuritis associated with adalimumab in the treatment of uveitis. *Ocular Immunology and Inflammation*.

[48] Theibich A., Dreyer L., Magyari M., Locht H. (2014). Demyelinizing neurological disease after treatment with tumor necrosis factor alpha-inhibiting agents in a rheumatological outpatient clinic: description of six cases. *Clinical Rheumatology*.

[49] Gregory A. P., Dendrou C. A., Attfield K. E. (2012). TNF receptor 1 genetic risk mirrors outcome of anti-TNF therapy in multiple sclerosis. *Nature*.

[50] De Jager P. L., Jia X., Wang J. (2009). Meta-analysis of genome scans and replication identify CD6, IRF8 and TNFRSF1A as new multiple sclerosis susceptibility loci. *Nature Genetics*.

[51] Tuller T., Atar S., Ruppin E., Gurevich M., Achiron A. (2013). Common and specific signatures of gene expression and protein-protein interactions in autoimmune diseases. *Genes and Immunity*.

[52] Bielekova B., Komori M., Xu Q., Reich D. S., Wu T. (2012). Cerebrospinal fluid IL-12p40, CXCL13 and IL-8 as a combinatorial biomarker of active intrathecal inflammation. *PLoS ONE*.

[53] Samavedam U. K. S. R. L., Kalies K., Scheller J. (2013). Recombinant IL-6 treatment protects mice from organ specific autoimmune disease by IL-6 classical signalling-dependent IL-1ra induction. *Journal of Autoimmunity*.

[54] Windhagen A., Anderson D. E., Carrizosa A., Balashov K., Weiner H. L., Hafler D. A. (1998). Cytokine secretion of myelin basic protein reactive T cells in patients with multiple sclerosis. *Journal of Neuroimmunology*.

[55] Hohnoki K., Inoue A., Koh C.-S. (1998). Elevated serum levels of IFN-*γ*, IL-4 and TNF-*α*/unelevated serum levels of IL-10 in patients with demyelinating diseases during the acute stage. *Journal of Neuroimmunology*.

[56] Kidd P. (2003). Th1/Th2 balance: the hypothesis, its limitations, and implications for health and disease. *Alternative Medicine Review*.

[57] Ahn J., Feng X., Patel N., Dhawan N., Reder A. T. (2004). Abnormal levels of interferon-gamma receptors in active multiple sclerosis are normalized by IFN-*β* therapy: implications for control of apoptosis. *Frontiers in Bioscience*.

[58] Furlan R., Bergami A., Lang R. (2000). Interferon-*β* treatment in multiple sclerosis patients decreases the number of circulating T cells producing interferon-*γ* and interleukin-4. *Journal of Neuroimmunology*.

[59] Serra C., Mameli G., Arru G., Sotgiu S., Rosati G., Dolei A. (2003). In vitro modulation of the multiple sclerosis (MS)-associated retrovirus by cytokines: implications for MS pathogenesis. *Journal of NeuroVirology*.

[60] Skurkovich S., Boiko A., Beliaeva I. (2001). Randomized study of antibodies to IFN-*γ* and TNF-*α* in secondary progressive multiple sclerosis. *Multiple Sclerosis*.

